# The WHO-5 well-being scale and its correlation to depressive and manic symptoms among outpatients with bipolar disorder or unipolar depression

**DOI:** 10.1192/j.eurpsy.2021.232

**Published:** 2021-08-13

**Authors:** S. Straszek, A.-E. Christensen, R. Licht, S. Østergaard, R. Ernst Nielsen

**Affiliations:** 1 Research And Treatment Programme For Bipolar Disorder, Psychiatry - Aalborg Úniversity Hospital, Aalborg, Denmark; 2 Department Of Clinical Medicine, Aalborg University, Aalborg, Denmark; 3 Unit For Psychiatric Research, Psychiatry - Aalborg University Hospital, Aalborg, Denmark; 4 Department Of Clinical Medicine, Aarhus University, Aarhus, Denmark; 5 Department Of Affective Disorders, Aarhus University Hospital - Psychiatry, Aarhus, Denmark; 6 Psychiatry, Aalborg University Hospital, Aalborg, Denmark

**Keywords:** bipolar disorder, who-5, quality of life, unipolar depression

## Abstract

**Introduction:**

There is a lack of longitudinal studies of patients with bipolar disorder (BD) or unipolar depression (UD) in terms of psychological well-being as measured by the WHO-5 and the correlation to symptom scores. It is of interest to investigate whether the WHO-5 is useful in monitoring patients with mood disorders over time, as a tool in measurement-based care, and as a supplement to other psychometric measures.

**Objectives:**

In this study we investigate the correlation at baseline between the depressive symptom scores according to the 6-item Hamilton Depression Score (HDS-6) and the WHO-5 scores in outpatients treated for BD or UD. Furthermore, in patients with BD we investigate correlations between manic symptom scores according to the modified Bech-Rafaelsen Mania Scale (MAS-M) and the WHO-5 scores. Lastly, in patients with BD or UD, we investigate the correlations between endpoint-baseline change in WHO-5 and change in MAS-M and HDS-6.

**Methods:**

A longitudinal study of 200 outpatients diagnosed and treated for either BD or UD. Patients will be measured at baseline and at least four weeks later. Baseline data are presented as frequencies, means and standard deviations or medians with interquartile ranges as appropriate. All correlations are presented as scatter plots and a Spearman correlation analysis

**Results:**

The study is ongoing, but the results will be available for presentation at the EPA in 2021.

**Conclusions:**

The WHO-5 may represent a relevant outcome measure in the treatment of BD and UD.

**Disclosure:**

No significant relationships.

